# Risks and uncertainty associated with prices set by equine boarding facility owners

**DOI:** 10.1093/tas/txaf107

**Published:** 2025-08-19

**Authors:** Jada M Thompson, Michelle L Kibler, Jennie L Z Ivey

**Affiliations:** University of Arkansas, Agricultural Economics and Agribusiness Department, Fayetteville, AR 72701, USA; Illinois State University, Department of Agriculture, Normal, IL 61761, USA; University of Tennessee, Animal Science Department, Knoxville, TN 37996, USA

**Keywords:** boarding rate, equid boarding, equine facility owners, equine housing, stabling, willingness-to-pay

## Abstract

Equine boarding facilities provide critical care for a large portion of the U.S. equine population, yet rising input costs challenge facility owners’ ability to maintain services without adjusting fees. Understanding how facility owners perceive boarders’ willingness to pay (WTP) for increased boarding costs is essential for balancing equine welfare with business sustainability. This study aimed to 1) assess equine facility owners’/managers’ perceptions of how boarders would respond to increased boarding rates, and 2) identify factors influencing those perceptions. A 26-question online survey targeting equine boarding facility owners and managers (n = 112 completed responses) was distributed via Extension channels and social media between July and August 2020. Facility owners reported average monthly board rates and answered a double-bounded dichotomous choice WTP scenario based on a hypothetical increase in care costs. Interval regression analysis was used to estimate perceived WTP, controlling for facility size, boarding type, region, income, and demographic factors. Overall, facility owners perceived that boarders would tolerate a 14.28% (P < 0.01) increase in monthly boarding rates. Full board clients were perceived to have slightly lower WTP (13.42%, P < 0.05); however, due to higher baseline costs, this translated into a greater absolute monthly increase ($69.78) compared to other board types ($34.85). Owners with higher household incomes perceived greater boarder WTP (P < 0.05), while facility size and geographic region were not significant factors. These results suggest that although equine owners may tolerate modest rate increases, larger absolute increases for full board may require additional justification through service value. Understanding owner perceptions of boarder WTP can help boarding facilities make informed pricing decisions that support both business viability and equine welfare.

## Teaser

As costs for equine care rise, how much more are owners willing to pay to board their animals? A new study finds that boarding facility owners estimate boarders will tolerate a 14% increase in monthly fees. Understanding this balance is critical to keeping boarding facilities economically viable with reasonable expectations for boarders as equine care prices fluctuate.

## INTRODUCTION

With over 2.4 million equids in the United States and only 370,000 equine-dedicated farms, many equine owners choose to board or pay for offsite care and shelter for their animals (USDA, 2022). While the USDA officially classifies equids as livestock, equine owners may perceive their animals in diverse ways, ranging from livestock to companion animals, and equids may be utilized for production to recreational purposes (Hemsworth et al., 2014; Wallace et al., 2019; USDA, 2022; Thompson, et al., 2023). Varied perceptions and usage influence welfare expectations and care standards, particularly in the context of boarding services and pricing, which must cater to the needs of owners and equids alike (Melvin et al., 2020).

Equine boarding facilities provide essential and, in some instances, nonessential care to equids for an agreed upon rate (referred to as board rate or board cost), often collected on a monthly basis (Thompson et al., 2023). Concerns over facility operational cost among individuals who own or managed a minimum of one equid reported increases in barn supplies costs from 12% to 22% from 2018 to 2021 (American Horse Publications., 2018, 2021). Facility owners manage the daily care and associated costs, and are frequently challenged with offering the same level of care for a consistent fee despite changes in price for required items (e.g., forage, feed, bedding). While equine facilities may try to minimize production cost increases by purchasing alternative or bulk products or adjusting management practices, mitigating the increased cost of equine care may be accomplished through augmented boarding rates. Uncertainty of equine owner sensitivity and response to boarding rates is a large challenge for boarding facilities. Therefore, the need to balance rising costs with the potential loss of boarders if rates increase above boarder willingness-to-pay is paramount. To this end, understanding what equid owners are willing to pay in increased boarding rates is essential for continued success of facilities.

Thus, this study aims to 1) investigate equine facility owner/manager perceptions of how equine owners (boarders) would respond to an increase in boarding rates and 2) examine the factors that contribute to boarding facility owners’ perception of boarders’ willingness to pay (WTP) for services.

## MATERIALS AND METHODS

### Survey Development, Distribution, and Content

A 26-question online survey was first piloted in small sample of horse owners (n = 5) to adjust language and ensure clarity; these responses were not used in the analysis. It was then disseminated to equine owners and facility operators through developed email lists, state equine Extension specialists and county Extension agents, and social media (e**.**g**.,** Facebook, Instagram, Twitter) platforms of equine owners, leasers, and boarding facility owners (Qualtrics, Provo, UT). The study was reviewed and approved by the University of Tennessee Institutional Review Board (IRB-20-05940-XM). Participation in the survey was voluntary, limited to one response per IP address, and data collection spanned 4 wk from July to August 2020. Respondents were asked to self-identify as a facility operator, and utilizing skip logic, a series of questions were presented to assess their perception of equine owner’s WTP for boarding services.

Facility owner respondents were asked to report the number of equids they boarded for others using pre-determined population ranges: 0, 1, 2 to 4, 5 to 9, 10 to 19, or 20 or more, where individuals who responded with “0” horses boarded were not considered in as a boarder operator for this analysis. A free response question was then presented for respondents to share the approximate value they charge for monthly board, and if the majority of their clients pay for full, partial, or other boarding types (free response option provided for “other”). To prevent misinterpretation or personal bias, boarding definitions were provided, where partial board involved some cost for facility use, feed, or daily care (excluding veterinary and farrier costs) and full board covered all costs for facility use, feed, and daily care (excluding veterinary and farrier costs). Due to limited boarder operator responses, we regrouped categorial data collected into binary variables, i.e., where there were too few observations (i.e., fewer than 15 observations), responses were re-grouped into binary variables (Table 1).

**Table 1.** Summary of facility owner survey responses used in statistical analysis (N = 112)

Variable	Description	Mean	Std. dev.	Min	Max
Facility WTP^2^ % Low	Lower bound used in interval regression	11.02	4.67	1	20
Facility WTP^2^ % Upper	Upper bound used in interval regression	13.19	5.00	2	20
Boarder Costs	Monthly cost per equid at the facility	446	282	100	2,000
Full Board	1 if cost represents full board, 0 otherwise	0.73	0.44	0	1
Number Boarded: 1	1 if boarding capacity 1 equids, 0 otherwise	0.29	0.45	0	1
Number Boarded: 2 to 4	1 if boarding capacity 2 to 4 equids, 0 otherwise	0.27	0.44	0	1
Number Boarded: 5 to 9	1 if boarding capacity 5 to 9 equids, 0 otherwise	0.22	0.42	0	1
Number Boarded: 10 to 19	1 if boarding capacity 10 to 19 equids, 0 otherwise	0.14	0.35	0	1
AHHI^3^: Less than $50,000	1 if annual household income of respondent, 0 otherwise	0.25	0.44	0	1
AHHI^3^: $50,001 and $75,000	1 if annual household income of respondent, 0 otherwise	0.16	0.37	0	1
AHHI^3^: $75,001 and $100,000	1 if annual household income of respondent, 0 otherwise	0.21	0.41	0	1
AHHI^3^: $100,001 and $150,000	1 if annual household income of respondent, 0 otherwise	0.21	0.41	0	1
AHHI^3^: Greater than $150,000	1 if annual household income of respondent, 0 otherwise	0.17	0.38	0	1
South	1 if respondent location^1^	0.78	0.42	0	1
Age: Younger than 35	1 if respondents younger than 35 yr old	0.24	0.43	0	1

^1^Respondents residing in the South as defined by the U.S. Department of Commerce’s Census Regions (2013).

^2^Willingness-to-pay.

^3^Annual Household Income.

Facility owner annual gross household income was collected (less than $25,000, $25,001 to $50,000, $50,001 to $75,000, $75,001 to $100,000, $100,001 to $150,000, and greater than $150,000) to account for how respondent’s preconceived expectations from their own income may influence their cost of care expectations. As 78% of respondents resided in southern states and to assess regional effects, respondent’s reported state of residence was reclassified into a binary variable to represent facilities operating in the southern United States as defined by the U.S. Department of Commerce (2013). Because there were too few respondents by age categories in the survey, we created a binary variable for respondents younger than 35 yr old, 0 otherwise. Robustness checks confirmed no additional information was gained by separating the ages into more categories (Stata 18, 2023). Similar robustness checks were used for other variables as well where no additional information was gained by further separating variables into more categories.

### Willingness-To-Pay and Model Development

Facility owners were asked to evaluate a hypothetical situation where operational cost increased, and how they believed their current boarders would respond to a boarding cost increase. Using each boarding owner’s previously reported monthly rate for a single equid, a double bounded, dichotomous choice question was asked to elicit what the facility owner perceived their boarders’ WTP for increased cost of care, starting with a 10% increase in current care cost (*C*_*1*_) followed by a randomly assigned change (either 1% to 9% or 11% to 20%) based on the response to the first question ($C_{2}^{Y}$ and $C_{2}^{N}$) (Hanemann et al., 1991; Kibler and Pendell, 2018; Thompson et al., 2023). The random percent was selected using the Mersenne Twister algorithm in Qualtrics (2015) similar to Campbell et al. (2021) and Thompson et al. (2023). Flow pattern for sequence intervals and the proportion of facility owners’ responses (Table 2), along with random change in cost percent shown to each respondent was collected and used to create intervals for the statistical analysis. The mean upper bound was 13.19% boarding rate increase and the mean lower bound was 11.02% boarding rate increase (Table 1).

**Table 2.** Willingness to pay response sequence intervals and percent of survey responses

	Lower Bound	Upper Bound	% of Respondents
Yes, Yes	$C_{2}^{Y}$ : 11% to 20%	.	41%
Yes, No	C_1_: 10%	$C_{2}^{Y}$ : 11% to 20%	32%
No, Yes	$C_{2}^{N}$ : 1% to 9%	C_1_: 10%	15%
No, No	.	$C_{2}^{N}$ : 1% to 9%	13%

Note: Unrestrained values are presented as periods.

### Willingness to Pay and Statistical Analysis

To determine the facility owners’ perceived boarder WTP, an interval regression analysis of the double bounded responses was estimated in Stata 18 (2023). Interval regression uses intervals rather than an exact value for the dependent variable which allows for flexibility in interval censored data and incorporates uncertainty into the WTP estimations. This approach allows for a better estimate as respondents narrow their WTP. The log-likelihood function (LLF) for the interval regression can be simplified to the following, assuming a standard normal distribution function (Φ):


$$\begin{array}{l} LLF=ln\left[\Phi \left(\frac{C_{i,upper-X_{i}\beta }}{\sigma }\right)-\Phi \left(\frac{C_{i,lower-X_{i}\beta }}{\sigma }\right)\right] \end{array}$$ (1)

where the costs presented represent the upper and lower bounds (*C*_*i,j*_) as presented in Table 2 with standard error σ. The independent variables ($X_{i}$), as previously discussed, include type of cost provided (full board), facility capacity (number of equids boarded), and facility owner/manager demographics (i.e., location, age, annual household income). Using the estimated coefficients and mean values for the explanatory variables, the mean WTP was determined (Tonsor et al., 2013; Thompson et al., 2023). Robust standard errors were used, which use White’s standard errors to correct for heteroskedasticity in the data.

## RESULTS

Respondents were all facility owners (n = 112) and primarily managed 1 equid (37%, 41 respondents), with the next largest group boarding 2 to 4 equids (27%, 30 respondents). Facility owners (n = 112) indicated full board as the primary board classification for equine board at their facility (n = 81, 73%, Table 1). The mean cost of all boarding care was reported as $446 ± 282 per month but varied widely from $100 to $2,000 across all respondents. The average cost for full board was $520 ± 297 (n = 91) and $241 ± 98 (n = 33) for all other boarding types (Table 3). Overall, facility owners perceived that boarders would be willing to pay 14.28% (p < 0.01) more for monthly board.

**Table 3.** Facility owner boarder willingness-to-pay interval regression results (N = 112)

	Coefficient	Robust SE
Boarder Costs	0.06**	(0.02)
Full Board	13.42**	(5.90)
Full Board × Boarder Costs	−0.06**	(0.02)
Number Boarded: 2 to 4	−2.47	(1.78)
Number Boarded: 5 to 9	−0.20	(1.94)
Number Boarded: 10 to 19	−1.43	(2.22)
AHHI^1^: $50,001 and $75,000	3.46	(2.21)
AHHI^1^: $75,001 and $100,000	4.66**	(2.25)
AHHI^1^: $100,001 and $150,000	3.81	(2.52)
AHHI^1^: Greater than $150,000	8.66^***^	(2.86)
South	0.64	(1.79)
Age less than 35	1.80	(1.89)
Constant	−2.45	(6.37)
Expected Willingness-to-Pay (%)	14.28^***^	(0.45)
Ln (Sigma)	1.86^***^	(0.11)

^***^p < 0.01, ** p < 0.05.

^1^Annual Household Income.

Boarder costs were significant factors in WTP, but this differed by boarding type. For full board, increased boarding rates decreased WTP by −0.06% (p < 0.05). Full board is more expensive, and while the relative increase is comparable to other boarding types, the absolute rate increases would vary resulting in larger amounts paid by individuals seeking full board over other boarding types. In general, full boarders have a higher WTP (13.42%, p < 0.05) but this is mitigated by their absolute boarding cost effects. For example, at their means and holding all else constant, a full boarder would pay 13.42% compared to other boarding types WTP 14.46%, but these percentages are based on base care rate such that in this scenario the actual increase for full boarders would be $69.78 per month per equid compared to $34.85 for other boarding types.

Income of the facility owners influenced their perception of boarder WTP. Facility owners earning $75,001 and $100,000 per year and more than $150,000 both perceived a higher WTP, 4.66% (p < 0.05) and 8.66% (p < 0.01), respectively as compared to those earning less than $50,000 per year. The highest amount expected that the market would bear were from respondents in the highest bracket.

## DISCUSSION

This study provides novel information on facility owners and perceived tolerance of boarding cost increase within the market. Understanding there are additional factors influencing boarding decisions for equine operations, the consistency between facility owners’ perception and boarders WTP may indicate that prices and the market are well understood, even in the middle of financial instability. Total expected WTP from this study was 14.0%, which is consistent with a boarding survey by Thompson et al. (2023) where boarders’ WTP was 14.2% higher than their reported monthly boarding rates. This continuity of expectations on what the market could bear reflects market efficiency despite a lack of a centralized information source. Further, results could reflect the integrated relationship between facility owner and boarder, where boarders are heavily involved in the cost of care and understand the prevailing markets.

Equid facility manager/owner perceptions of how much increased board rates equid owners would pay is reflective of the market scope during the study data collection period (July-August 2020). Equid owners may have been WTP more or less because of the ongoing pandemic and resulting uncertainty, or these findings might be consistent with their true WTP regardless of the pandemic. Thompson et al. (2023) found equid owners relatively unimpacted financially during the early stages of the pandemic with only 3% of respondents indicating they were “*less financially stable*” as a result of the pandemic. The total number of equids transported by air during the pandemic decreased but were found to be statistically insignificant (Felici et al., 2023) indicating no relevant change to this sector of the equine industry. In the Kentucky equine market, minimal impacts to breeding operations and boarding/training/lesson operations were identified due to the pandemic, with the largest impact to competition operations and facilities (Stowe et al., 2021). Prior to the pandemic, interest in virtual competitions or horse shows was increasing, and with an average of 5 missed in-person competitions due to the pandemic, virtual competitions/shows may have served as a less expensive alternative (Walker et al., 2022). Thus, despite timeframe contingencies on WTP results from this study, data provided are valuable and relatable to the currently industry’s economic stability and equine industry resilience post-COVID.

Boarding facility owners and managers facing rising input costs must either absorb the costs, adjust services provided, or pass these costs on to boarders through increased board rates and fees. Raising rates can be a difficult decision if facility owners are unsure of how boarders will respond to changes in prices and services. Boarding facilities must balance the need to cover costs with maintaining an optimal number of equids boarded to remain profitable.

Boarding agreements or contracts may help mitigate boarding cost concerns by providing details on boarding fee breakdown and aid in determining WTP for essential and non-essential services. Boarding contracts are legal documents signed by both the equine facility and the equid owner to outline the services to be performed by the facility (daily feedings, turnout, etc.) and those not covered by the boarding rate or available for an additional fee (e.g., veterinary expenses, farrier expenses, blanketing, etc.). Contracts are designed to protect the facility and facility ownership in the event of death, injury, or illness to the equid, failure to remit board rates to the facility by the equid owner, or any other situation which puts the facility and/or facility management at risk; however, contracts may be intermittently utilized or updated (Porr and Childs, 2008). Although not a component of this study, characteristics and utilization of boarding contracts across varied facility types and boarding levels may prove beneficial in understanding WTP among equine owners.

Equine facility managers/owners have numerous responsibilities including care for the equids they manage (equid wellbeing), successfully maintaining a facility, managing client expectations, meeting policy requirements (manure management, conservation practices, etc.), and interactions with the non-equine community surrounding the facility (Furtado et al., 2021; Zagonel et al., 2021). Additionally, facilities face rising costs of management due to supply chain disruptions, environmental issues affecting forage and feed costs, and other product sourcing challenges. Many equine operations prioritize equid welfare (Furtado et al., 2021) when managing increase cost of equid care or the expectations of increased costs on equine owner WTP. Concern over horsekeeping cost among individuals who own or managed a minimum of one equid was found in the 2018 and 2021 Equine Industry Surveys where feed was reported most often as the primary area of increased cost of equine ownership while 42% indicated cost of maintaining horses to be a major area of concern to the equine industry (American Horse Publications 2021). Survey respondents reported increases in barn supplies costs from 12% to 22% while 22% of those surveyed indicated fuel expense to be the primary source of increased cost (American Horse Publications). Equine facility owners are challenged with offering quality care while the costs for that level of care (e.g., forage, feed, bedding) rises. While equine facilities may try to minimize production cost increases (purchasing alternative products, adjusting management practices, taking advantage of bulk pricing, etc.), another method for mitigating the increased cost of equine care is through augmented board rates. Uncertainty of equine owner sensitivity and response to boarding rates is a large challenge for boarding facilities. Therefore, the need to balance rising costs with the potential loss of boarders if rates increase above boarder willingness-to-pay is paramount.

Boarder willingness to absorb some or all of increasing input costs of facilities is likely due, in some part, to available substitutes, or the proximity of adequate boarding facility options for their equid(s). Equine owners require specialized facilities designed for equids given the housing requirements specific to equids and the desired use of the animal. It is possible that boarding facility amenities (riding arenas, turnout options, add-on services) and an owner’s emotional connection to their equid(s) plays a role in boarder WTP and may influence perceived adequacy of available substitutes. Further, data indicating the highest market bearing board increase is from individuals with higher annual gross household income could reflect a larger disposable income, resiliency to rising costs, specialized boarding facility for niche/high value horses, and/or predisposition to boarding facility management of clientele at facilities owned by this subset of survey respondents. The facility capacity did not have a statistically significant impact on WTP, which may be explained by the fact that all costs are per equid per month. Often the size (capacity and/or acres) of a facility will matter in terms of economies of scale, which reduce the average costs per unit, however with a limited number of equids under care, the size of the facility did not play a statistically significant role in the perception of boarders WTP. Future work could investigate the density of boarding facilities in a given area, facility capacity, and respective impact on WTP for boarding services.
